# Trisomy 11 as an Additional Chromosome Alteration in a Child with Acute Promyelocytic Leukemia with Poor Prognosis

**DOI:** 10.1155/2012/659016

**Published:** 2012-07-05

**Authors:** Elenice Ferreira Bastos, Lidiane Alice Silva, Marcelo Coelho Ramos, Glicínia Pimenta, Paulo Ivo Cortez, Stella Beatriz Gonçalves de Lucena, Teresa de Souza Fernandez

**Affiliations:** ^1^Faculty of Medical Sciences, State University of Rio de Janeiro, 20551-120 Rio de Janeiro, RJ, Brazil; ^2^Bone Marrow Transplant Center, National Cancer Institute (INCA), 20551-120 Rio de Janeiro, RJ, Brazil; ^3^Serviçio de Hematologia, Hospital Universitário Pedro Ernesto, Avenue 28 de Setembro no. 77, 3*º* andar, Vila Isabel, 20551-120 Rio de Janeiro, RJ, Brazil; ^4^Hematology Service, HUCFF, UFRJ, 20551-120 Rio de Janeiro, RJ, Brazil; ^5^Pronto Baby Children's Hospital, 20551-120 Rio de Janeiro, RJ, Brazil

## Abstract

The prognostic significance of the additional abnormalities to the t(15; 17) remains controversial. We report a case of promyelocytic leukemia (APL) in a ten-year-old boy. Classical and molecular cytogenetic (FISH) studies of a bone marrow sample obtained at diagnosis revealed the presence of trisomy of chromosome 11 as an additional chromosomal abnormality to the t(15; 17). The presence of the translocation t(15; 17), the cytogenetic marker of APL, is usually associated with good response to treatment with ATRA. In this case, although the patient had risk factors associated with good prognosis, he evolved and died quickly. So it seems that the presence of the trisomy 11 may be associated with disease progression and the poor outcome. To our knowledge, this is the first reported case of t(15; 17) associated with trisomy of chromosome 11 in a child with APL.

## 1. Introduction

Acute promyelocytic leukemia (APL) is a specific type of acute myeloid leukemia (AML) characterized by the morphology of blast cells (M3 in the French-American-British classification) and presence of the t(15; 17)3 translocation. This translocation reflects the molecular rearrangement of the promyelocytic (*PML*) gene, located at 15q22, with the retinoic acid receptor-alpha (*RARA*) gene, located at 17q21, and it is considered as critical for the disease's pathogenesis since it blocks differentiation during the promyelocytic stage of myeloid maturation [1].

Immediate identification of t(15; 17) or the *PML/RARA* gene rearrangement is fundamental for the treatment. Treatment with all-transretinoic acid (ATRA) alone may induce complete remission in more than 80% of cases, but when combined with chemotherapy, it leads to significant improvement in survival rate and lowers the incidence of relapse [1]. Chromosomal rearrangements, in addition to t(15; 17), have been reported in 25–40% of APL patients. Trisomy 8 is the most frequent secondary anomaly, and other abnormalities involving chromosomes 9, 17, 21, 16, 6, and 12 have been described with less frequency [2]. The prognostic value of chromosomal abnormalities besides t(15; 17) remained uncertain in previous studies.

We described a case of APL in a child with trisomy 11 and t(15; 17) characterized by conventional and molecular cytogenetic analysis and reviewed the prognostic significance of chromosomal abnormalities in addition to t(15; 17) in APL.

## 2. Case Report

A 10-year-old boy was referred in May 2007 with hematomas without bleeding signals. Laboratory studies revealed a white blood cell (WBC) count of 2.9 × 10^9^/L, Hgb 11.6 g/dL, and platelets 29 × 10^9^/L with 43% of blast cells. The bone marrow aspirate was hypercellular, 84% of nucleated cells were blasts with granules, and occasionally Auer rods could be observed.

**Figure 1:**
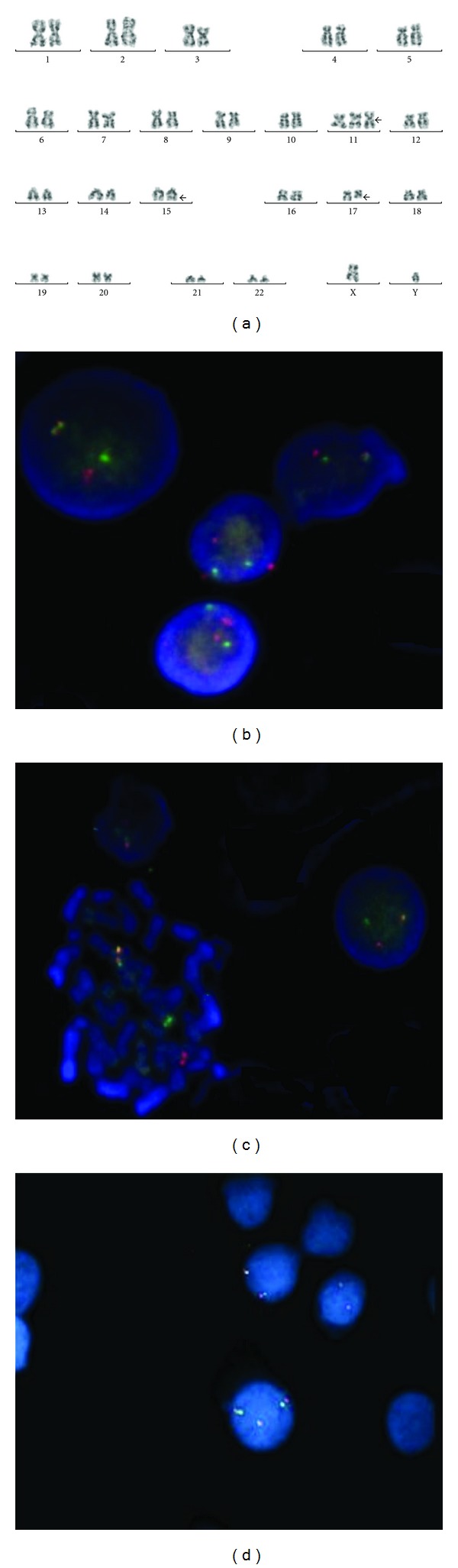
(a) Karyotype of bone marrow cell by G-banding showing 47,XY,+11,t(15; 17)(q22; q21). (b, c) Hybridization of LSI *PMLRARA *dual-color DNA probe (Cytocell, Cambridge, UK). The probe hybridizes to chromosome 15q11 (SpectrumOrange *PML*), 17q21.1 (SpectrumGreen RARA). Fusion signal of PML/*RARA* gene was clearly observed in 70% of the nucleus and metaphases. (d) Fish using LSI MLL dual color break-apart probe. Nucleus with three and others with two signals, confirming trisomy 11 in some of them.

Flow cytometry was performed on bone marrow cells using a panel of directly conjugated antibodies (Becton and Dickinson, San Jose, CA), and it showed the expression of CD45, CD13, CD33, and CD117 and low expression of CD34, HLA-DR, and T and B lymphocytic markers was negative. Karyotypes of bone marrow (BM) cells were obtained at the time of diagnosis. Cells were cultured for 24 hours without mitogenic stimulation. Chromosome preparation was made after a colcemid pulse (final concentration 10^6^ M) during the last hour of incubation; cells were processed by standard procedures. GTG banding was performed, and chromosomes were identified and analyzed according to the International System of Human Nomenclature [3] that showed the karyotype: 47,XY,+11,t(15; 17)(q22; q21)[17]/46,XY[3] (Figure 1(a)). We performed the FISH analysis to confirm the *PML/RARA *gene  rearrangement involving the t(15; 17)(q22; q21) and trisomy of chromosome 11 (Figures 1(b) and 1(c), resp.). The FISH analysis with specific *PML/RARA* fusion signal on derivative chromosome 15 was clearly observed in 70% of the nucleus and metaphases observed, confirming the 15; 17 translocation. The extra chromosome 11 was also observed in 70% of nucleus and metaphases analyzed. It was analyzed 200 cells (interphase nucleus and metaphases). The patient was treated with BFM 98 protocol for AML with all transretinoic acid achieving complete remission. Induction therapy with cytarabine, etoposide, and idarubicin was performed. The first consolidation was performed with cytarabine and mitoxantrone, and the second one was performed with cytarabine and mitoxantrone, and intensification with cytarabine, and etoposide. After this phase, it was done radiotherapy for prevention of central nervous system relapse and it was initiated maintenance with 6-tioguanine and cytarabine during four days a month until complete one year and a half of treatment. Nine months after treatment, the patient showed relapse of disease in bone marrow. The immunophenotypic, conventional, and molecular cytogenetic analysis confirmed this disease relapse. It was restarted the treatment with ATRA and BMF protocol, but the patient died two months after the resumption of treatment.

## 3. Discussion

The APL is around 10% of all types of acute myeloid leukemia (AML) diagnosed in children, representing 1% of leukemias in children. It is more common in children among ages of two and three years and in adults over 40 years. However, it can also be found in older children and teenagers [5]. The presence of the translocation t(15; 17), the cytogenetic marker of APL, is usually associated with good response to treatment with ATRA, and thus, it has been related with a favorable prognosis. The rapid diagnosis and immediate initiation of specific treatment are extremely important because of the high risk of early death in APL, as a consequence of bleeding. The morphological appearance of dysplastic hypergranulars promyelocytic allows the identification of typical cases, which, in itself, justifies the immediate start of treatment with ATRA. However, the subsequent conventional cytogenetic and FISH analysis at diagnosis for confirmation of APL is essential; they allow clarification of cases with atypical morphology and characterization of the translocation *PML*/*RARA* and variant translocations not involving *PML *gene, which usually has been associated with resistance to ATRA [6]. The cytogenetic characterization also allows the identification of additional chromosomal alterations besides t(15; 17) that can somehow influence the response to treatment. According to the Southwest Oncology Group (SWOG), the t(15; 17) with or without secondary chromosomal abnormalities has been associated with favorable prognosis [7]. Nevertheless, the prognostic significance of the additional abnormalities remains controversial. Several studies have shown that secondary chromosomal abnormalities do not influence therapeutic response and outcomes for newly diagnosed APL patients [2, 8]. Other report has shown that the presence of additional chromosomal changes adversely affects prognosis [9, 10]. To our knowledge, this is the first reported case of t(15; 17) associated with trisomy of chromosome 11 in a child with APL. In this case, the bone marrow had the typical morphologic appearance of APL usually seen with t(15; 17). Complete remission was achieved by treatment with ATRA. After one year, the patient showed relapse of disease, and it was restarted the treatment, but the patient evolved the disease and died after two months. Trisomy 11 as a sole chromosomal abnormality occurs with a frequency of 6-7% of the karyotypically aberrant hemopoietic neoplasms [1]. Slovak et al. reported a case of t(15; 17) with duplication of q23 region of chromosome 11 and associated this cytogenetic alteration with extremely poor response to chemotherapy [11]. Zhou et al. had associated trisomy 11 and the CD34 immunophenotype suggesting that trisomy 11 leukemia is characterized by a stem/progenitor cell immunophenotype with poor response to standard chemotherapeutic regimens and unfavorable prognosis. In this study, the authors suggested some possible mechanisms associated with trisomy 11 and poor prognosis: (a) a simple gene dose effect whereby the product of genes localized to chromosome 11 results in abnormal cell proliferation with or without imprinting consequences (b) duplication of a mutated proto-oncogene, or (c) nondisjunction secondary to primary molecular events resulting in uncontrolled cellular proliferation. The latter two possibilities prompted a molecular investigation of the *MLL* (myeloid lymphoid leukemia) gene, localized to band 11q23, which has been implicated in the pathogenesis of several hematological neoplasias [12].

We suggest that trisomy 11 could represent a risk factor for poor outcome, because our patient showed factors associated with favorable prognosis such as younger age [8], hypergranular APL (typical AML-M3 according to FAB classification), and cytogenetic favourable risk according to the SWOG. Therefore, the trisomy 11 may be associated with disease progression, representing a cytogenetic clonal evolution. In our opinion, the influence of additional chromosomal changes in the patient's prognosis is directly related to the chromosomal changes and thus to the genes involved in these alterations. Further characterization of the molecular events of trisomy 11 in hematological malignancies is needed to understand its role(s) in the pathogenetic mechanisms leading to the development and progression of leukemia.
